# *Synechococcus* Under Stress: Contrasting Physiological and Transcriptional Responses to Salinity and Temperature in Marine Versus Euryhaline Strains

**DOI:** 10.1111/1758-2229.70273

**Published:** 2026-01-26

**Authors:** Isabel Escribano‐Gómez, Rebeca Pérez, Uxue Arrizabalaga, Raquel Liébana, Miriam Vergara‐Len, Ángel López‐Urrutia, Laura Alonso‐Sáez

**Affiliations:** ^1^ AZTI, Marine Research, Basque Research and Technology Alliance (BRTA) Sukarrieta Spain; ^2^ Centro Oceanográfico de Gijón/Xixón, Instituto Español de Oceanografía, IEO‐CSIC Gijón Spain

**Keywords:** salt acclimation, *Synechococcus*, temperature acclimation, transcriptomic analysis

## Abstract

Temperature and salinity are key environmental drivers that constrain growth and distribution of marine cyanobacteria, yet their combined physiological effects remain unexplored. We analysed the physiological and transcriptional responses of two *Synechococcus* strains, the marine RS9907 and euryhaline WH5701, across a salinity gradient (18–50 PSU) under optimal (28°C–30°C) and low temperature conditions (15°C–20°C). Growth and photosynthetic efficiency (*F*_V_/*F*_M_) declined under salinity stress (18 PSU for RS9907 and 50 PSU for WH5701), relative to typical marine conditions (36 PSU). RS9907 maintained the photosynthetic electron transport rate under salt stress and the *F*_V_/*F*_M_ under cold conditions more effectively than WH5701. Salinity induced a stronger regulatory response in WH5701 (71% genes differentially expressed, compared to only 6% in RS9907). Both strains shared a core response, upregulating carbon fixation genes under cold stress, and glycogen degradation and osmolyte synthesis genes at high salinity (42–50 PSU). Conversely, some photosynthetic genes (*psbCD*, *psaC*) showed increased expression at low salinity, but temperature‐dependent regulatory differences were observed. WH5701 uniquely upregulated genes related to membrane transporters, fatty acid desaturases and the pentose phosphate pathway within salinity, potentially contributing to their broader tolerance to salt fluctuations. Collectively, our results reveal contrasting strategies of thermohaline acclimation in *Synechococcus* strains adapted to different salinities.

## Graphical abstract

Two model strains of *Synechococcus* sp.—the marine RS9907 and the euryhaline WH5701—exhibit distinct physiological responses to salinity after long acclimations at optimal and cold growth temperatures. Transcriptomic and photochemical analyses reveal drastic differences in their regulatory strategies and photosynthetic activity across a salinity gradient, yet highlight shared expression patterns in key functional genes to cope with combined thermal and salinity stress.

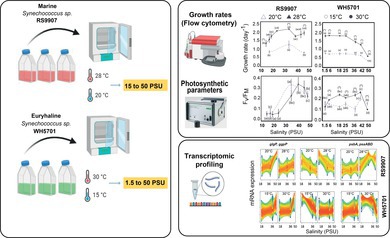

## 1 Introduction

Marine cyanobacteria contribute approximately 25% of the global net primary production and play a crucial role in regulating global biogeochemical cycles (Flombaum et al. 2013). The two major marine cyanobacterial lineages, *Prochlorococcus* and *Synechococcus*, exhibit contrasting distribution patterns across marine systems (Zwirglmaier et al. 2008). While *Prochlorococcus* preferentially thrive in warm oligotrophic oceanic areas, *Synechococcus* are more widely distributed in marine, brackish and freshwater ecosystems (Partensky et al. 1999; Xia et al. 2017; Cabello‐Yeves et al. 2022). Among *Synechococcus* lineages, the three major subclusters differ in their distribution patterns. Subcluster 5.1 consists of strictly marine species (except for the halotolerant clade VIII); subcluster 5.2 contains marine, brackish, euryhaline and freshwater isolates; and subcluster 5.3 includes both open ocean strains and a cosmopolitan group of freshwater strains (Cabello‐Yeves et al. 2017, 2018; Sánchez‐Baracaldo et al. 2019; Scanlan et al. 2009). Thus, *Synechococcus* have undergone multiple transitions between freshwater and marine environments throughout their evolutionary history (Sánchez‐Baracaldo 2015; Sánchez‐Baracaldo et al. 2019), likely driving the evolution of diverse adaptive strategies.

Variations in pigment composition have been considered a key feature for the adaptability of *Synechococcus* to a wide range of environmental conditions (Grébert et al. 2018). The significant diversity of pigmentation in *Synechococcus* strains is associated with the composition of their light‐harvesting antenna complex, that is, the phycobilisomes (Scanlan et al. 2009; Six et al. 2007). *Synechococcus* are classified into three pigment types: (i) those containing only phycocyanin (PC); (ii) those composed solely of phycoerythrin I (PEI); and (iii) those containing a combination of PC, PEI and phycoerythrin II (PEII; Six et al. 2007). *Synechococcus* strains belonging to the PC type exhibit a strong tolerance to variations in salinity, while those belonging to the PE (PEI, PEII), predominantly assigned to subcluster 5.1, are unable to grow in low salinity environments (Xia et al. 2015).

Most previous studies on salinity adaptation mechanisms in *Synechococcus* have focused on freshwater or estuarine strains (Alsamhary 2020; Chen et al. 2023; Fulda et al. 1999; Hagemann et al. 1994; Kanesaki et al. 2002; Ludwig and Bryant 2012; Marin et al. 2004; Xia et al. 2023), with very few targeting marine cyanobacteria (Cabello‐Yeves et al. 2022). At the cellular level, exposure to high salt concentrations initially results in a drastic decrease of cytoplasmic volume due to the loss of water and turgor pressure (Allakhverdiev and Murata 2008; Hagemann 2011; Klähn et al. 2021). The increase in intracellular concentration of ions, in turn, disrupts cellular constituents, including membrane proteins (Huang et al. 2006) and the photosynthetic apparatus (Allakhverdiev et al. 2000; Allakhverdiev and Murata 2008; Yang et al. 2020). However, *Synechococcus* exhibits mechanisms to protect from high salt stress, including the modulation of the expression of photosynthetic genes (Al‐Hosani et al. 2015; Allakhverdiev et al. 2002; He et al. 2022; Xia et al. 2020), the active extrusion of toxic inorganic ions (Allakhverdiev and Murata 2008; Hagemann 2011), and the synthesis of compatible solutes (Brown 1976; Hagemann 2011; Klähn and Hagemann 2011).

Compatible solutes are low molecular mass organic molecules, which are accumulated inside the cells without altering cellular metabolism (Brown 1976). Different compatible solutes are synthesised depending on the salt tolerance limit of cyanobacteria strains (Brown 1976; Hagemann 2011; Kirsch et al. 2019). Strictly freshwater strains, with a typical tolerance limit of 3–5 g/L of NaCl (≈3–5 Practical Salinity Unit, PSU), accumulate sucrose and/or trehalose. Euryhaline strains, with a tolerance limit up to ca. 48 g/L of NaCl (≈48 PSU), accumulate sucrose, trehalose and glucosylglycerol. Marine strains, that exhibit a salt tolerance limit above 35 g/L of NaCl (≈35 PSU), typically accumulate glucosylglycerol, glucosylglycerate and glycine betaine (Cabello‐Yeves et al. 2022; Hagemann 2011; Klähn and Hagemann 2011), while sucrose accumulation seems to be minor or only transient (Hagemann 2011; Kirsch et al. 2019). At the molecular level, the induction of genes involved in the synthesis of compatible solutes represents a central component of the salt stress response in *Synechococcus* (Allakhverdiev et al. 1999, 2001; Cumino et al. 2010; Kirsch et al. 2019; Ludwig and Bryant 2012), and also in its sister clade *Prochlorococcus* (Al‐Hosani et al. 2015).

Another mechanism to counteract the impact of salinity stress is the modulation of membrane lipid unsaturation (Allakhverdiev et al. 1999, 2001; Allakhverdiev and Murata 2008; Sakamoto and Murata 2002; Yang et al. 2020). Fatty acid desaturases are enzymes that introduce double bonds into specific positions within fatty acyl chains, resulting in the formation of monounsaturated or polyunsaturated fatty acids (D. Los and Murata 1998; Murata and Wada 1995). Although these enzymes have been predominantly studied for their role in temperature acclimation of freshwater and marine cyanobacteria (Breton et al. 2020; Los and Murata 1999; Murata and Los 1997; Pittera et al. 2018; Varkey et al. 2016), some studies have also highlighted their involvement in the salt stress response in freshwater strains (Allakhverdiev et al. 1999, 2001). Indeed, freshwater and estuarine *Synechococcus* strains contain a larger number of fatty acid desaturases (*desA*, *desB*, *desC* and *desD*) than marine strains, which only harbour *desC* and *desA*. This has been related to their need to cope with greater variations in both temperature and salinity (Allakhverdiev et al. 1999, 2001; Los 2004; Pittera et al. 2018; Sakamoto and Murata 2002).

Beyond salinity, temperature also exerts a significant influence on the physiological and molecular responses of picocyanobacteria, revealing contrasting acclimation strategies among clades (Alonso‐Sáez et al. 2023; Labban et al. 2023; Mackey et al. 2013; Pittera et al. 2014; Varkey et al. 2016). Previous studies on the global molecular response of *Synechococcus* or *Prochlorococcus* to temperature or salinity have addressed these two parameters separately (Al‐Hosani et al. 2015; Alonso‐Sáez et al. 2023; Breton et al. 2020; He et al. 2022; Labban et al. 2023; Ludwig and Bryant 2012; Mackey et al. 2013; Marsan Wilfred 2016; Pittera et al. 2018; Varkey et al. 2016; Xia et al. 2020). However, it is known that environmental stressors can exert either synergistic or antagonistic effects on the growth of phytoplankton (Kholssi et al. 2023). Indeed, a potential interaction between salinity and temperature may underlie the puzzling observation that marine *Synechococcus* are unable to survive in polar marine waters, while they can reach high abundances in polar freshwater lakes (Powell et al. 2005).

Here, we performed an integrative analysis of the response to salinity and temperature acclimation in two *Synechococcus* sp. strains: (i) the strictly marine *Synechococcus* sp. RS9907 (subcluster 5.1, clade II), which is affiliated with the most abundant *Synechococcus* genotype found in the Tara Ocean dataset (Farrant et al. 2016), and (ii) the euryhaline *Synechococcus* sp. WH5701, a representative strain of subcluster 5.2, well adapted to broad salinity changes (Cabello‐Yeves et al. 2022; Fuller et al. 2003). We analysed the physiological response of these cyanobacteria to salinity conditions under both low and optimal growth temperatures in long acclimation experiments, to assess the sensitivity of these strains to combined stress conditions. Transcriptome sequencing (RNA‐Seq) was performed to get insights into the molecular basis for the thermohaline acclimation in marine and euryhaline *Synechococcus* strains.

## 2 Methods

### 2.1 Cyanobacterial Growth Conditions and Experimental Set‐Up

The non‐axenic marine cyanobacterium *Synechococcus* sp. RS9907 and the axenic euryhaline *Synechococcus* sp. WH5701 were used in our experimental work (referred to RS9907 and WH5701 hereinafter). RS9907 was obtained from the Roscoff Culture Collection (Roscoff, France), and WH5701 was provided by the National Center for Marine Algae and Microbiota, Bigelow Laboratories, East Boothbay, Maine. Cultures were grown in PCR‐S11 Red Sea Salt‐based medium supplemented with 1 mM sodium nitrate (Rippka et al. 2000). The original recipe of PCR‐S11 Red Sea Salt medium was modified by adding 40 g salt L^−1^ to obtain a salinity of 36 PSU, closer to marine salinity conditions (Antonov et al. 2010). The quantity of salt (in grammes per litre) was modified in accordance with the salinity range studied. Both *Synechococcus* strains were grown without agitation under continuous light provided by white light fluorescent tubes in temperature‐controlled chambers (ICO240 memmert, Memmert GmbH + Co. KG, Germany). RS9907 and WH5701 were grown under 50 and 30 μmol quanta m^−2^ s^−1^, respectively. Irradiance was measured with a quantum sensor (MQ‐500 full‐spectrum quantum metre with separate sensor, Apogee Instruments).

First, we acclimated both strains at cold and optimal growth temperature conditions (Figure S1). The thermal acclimation was longer in the case of the non‐axenic RS9907 strain (ca. 6 months) compared to the axenic strain WH5701 (3 weeks, Figure S2). Despite several attempts, we were unable to maintain axenic conditions for long‐term acclimations of the WH5701 strain, and therefore a shorter acclimation period was used. The growth temperature range of RS9907 had been reported in previous studies (Palacio et al. 2020; Labban et al. 2021; Escribano‐Gómez et al. 2025). Based on these previous results, we selected 20°C and 28°C as cold and optimal growth conditions for this strain, respectively. For strain WH5701, we characterised its thermal growth range in this study, as it had not been previously analysed. Thermal acclimation of WH5701 started from 24°C, which was the temperature of culture maintenance. Temperature was then adjusted along a range of temperatures between 11°C and 32°C. The lowest temperature (11°C) was identified as the cold temperature threshold limit for this strain, where no growth was detected under our experimental conditions. Based on the growth rate profile obtained, we selected 30°C as the optimal growth temperature and 15°C as a representative of cold temperature for WH5701.

During the salinity acclimation experiments, cultures were maintained in exponential growth by transferring them to fresh media. RS9907 cultures were initially grown at the optimal growth temperature (28°C) along a range of eight salinities: 15, 18, 20, 25, 36, 42, 47 and 50 PSU. At the low temperature condition of 20°C, we selected three contrasting salinities for comparison: 18, 36 and 50 PSU. The entire acclimation period for RS9907 cultures in each salinity and temperature condition lasted at least 6 months before RNA sample collection. In the case of WH5701, we acclimated the cultures to a broader range of salinities from 1.5 to 50 PSU, including 1.5, 6, 18, 25, 36, 42 and 50 PSU, under both cold and optimal growth temperatures for 3 weeks (i.e., at least 16 generations). During the acclimation procedure, cell abundance was monitored by flow cytometry (see below). Once full acclimation was achieved, we measured the growth rate and photosynthetic parameters (Figure S3), and RNA samples for gene expression analysis were collected. All samples were collected in at least three biological replicate cultures.

### 2.2 Flow Cytometry

To measure cell abundance during the acclimation experiments, samples were preserved with glutaraldehyde (ScharLab, S.L.) at a final concentration of 0.25%. Samples were incubated with the fixative for 10 min at room temperature and dark conditions and then stored at −80°C until processing. Unstained samples were analysed in a BD Accuri C6 Plus Flow Cytometer (BD, Biosciences), based on the natural fluorescence of the photosynthetic pigments of cyanobacterial cells (Marie et al. 1999), to quantify cell concentration. Maximum growth rates (μ, in day^−1^) were calculated as the slope of Ln (*Nt*) versus time during exponential phase, where *Nt* is the cell abundance at time *t*, using R statistical software.

### 2.3 Photochemistry Analysis

The photosynthetic measurements were carried out using a PHYTO‐PAM‐II Compact Version (Walz) and the PhytoWin_3 software for data acquisition. All measurements were conducted in living fresh cultures with a cell density of approximately 10^7^ cells mL^−1^. Starting with dark acclimated samples, 2 mL of culture were pre‐illuminated with far‐red light for 5 min to obtain a quasi‐dark state for *F*_V_/*F*_M_ determination. The *F*_V_/*F*_M_ (i.e., the maximum photochemical quantum yield of photosystem II, PSII) was obtained after applying a saturated light pulse. Subsequently, the *Y*(II), that is, the effective photochemical quantum yield of PSII, and the relative measure of electron transport rate (rETR) were determined by exposing the samples to pulses of actinic light with increasing intensity. The measuring wavelength was set at 540 and 590 nm for RS9907 and WH5701, respectively. The range of actinic light pulse was from 0 to 200 of photosynthetically active radiation (PAR) in μmol quanta m^−2^ s^−1^ units and each irradiance step was about 10 s.

The fluorescence parameters were calculated as following: (1) maximum PSII yield, $F_{V}/F_{M}=\frac{F_{M}-F_{0}}{F_{M}}$; (2) effective PSII yield $Y\left(\mathrm{II}\right)=\frac{F_{M}^{\prime }-F}{F_{M}^{\prime }}$; and (3) $\text{rETR}=Y\left(\mathrm{II}\right)\times \mathrm{PAR}\times P_{\mathrm{PS}2}/P_{\mathrm{PPS}}\times \mathrm{ETR}‐\text{Factor}$, where *F*_0_ is the minimum fluorescence level excited by very low intensity of measuring light, to keep PSII reaction centers open; *F*_M_ is the maximum fluorescence level after applying saturating light, which closes all PSII reaction centers; *F* is the fluorescence yield before the saturation pulse; *F*_M_′ is the maximum fluorescence level of the illuminated sample; *P*_PS2_/*P*_PPS_ is the ratio between photons absorbed by PSII relative to photons absorbed by all photosynthetic pigments, and ETR‐Factor is the ratio between photons absorbed by photosynthetic pigments and incident photons. An assumed value of 0.42 was used for the ETR‐Factor multiplied by *P*_PS2_/*P*_PPS_ in the rETR measurement according to manufacturer's instructions (Walz).

### 2.4 RNA Samples Collection and Extraction

For RNA sequencing analysis, cultures grown at 18, 36 and 50 PSU were initially chosen for comparing the response of RS9907 and WH5701. However, at the low temperature condition (15°C), the growth of WH5701 at 50 PSU was unstable and it could not be maintained during the whole acclimation period for obtaining samples for RNA sequencing. Thus, the 50 PSU condition was replaced by 42 PSU as a high salinity condition at 15°C for WH5701.

Exponentially growing cultures at a cellular density of approximately 10^7^ cells mL^−1^ were harvested by filtration using polyethersulfone membrane filters (0.2 μm pore size and 47 mm diameter, Sartorius) in Nalgene filter columns. Once filtered (50–100 mL in ≤ 2 min), samples were rapidly frozen in liquid N_2_ and stored at −80°C until RNA extraction.

Total RNA was extracted using the MirVana miRNA isolation Kit (Thermo Fisher Scientific) according to manufacturer's instructions. The eluted RNA was treated with Turbo DNase (Invitrogen by Thermo Fisher Scientific). RNA was quantified by a NanoDrop ND‐1000 spectrophotometer (NanoDrop Technologies Inc.) and Invitrogen Qubit RNA High Sensitivity (HS) kit. RNA quality and integrity was verified using the Agilent RNA 6000 Nano Bioanalyzer kit (Agilent Technologies). The absence of genomic DNA contamination was checked by PCR.

The RNA sequencing library was generated from 200 to 500 ng total RNA with the Illumina Stranded Total RNA Prep with Ribo‐Zero Plus Microbiome kit (Illumina). The cDNA libraries were sequenced as 50‐bp paired‐end reads on an Illumina NovaSeq 6000 sequencing platform at CNAG facilities (Spain).

### 2.5 Raw Data Processing

The quality of reads was checked with the FastQC tool (Bolger et al. 2014), and sequences were trimmed and paired using Trimmomatic (Bolger et al. 2014). We used the parameters SLIDINGWINDOW of 50:35 and MINLEN of 50 for trimming. SortMeRNA (Kopylova et al. 2012; Kopylova 2014) was used to filter rRNA fragments. The remaining reads were mapped against the genome of RS9907 (BV‐BRC^3.29.20^ Taxon ID 221350) and WH5701 (BV‐BRC^3.29.20^ Taxon ID: 69042) using bowtie2 (Langmead and Salzberg 2012; with ‘‐‐non‐deterministic’ parameter). Read count tables were obtained using HTSeq2 (Anders et al. 2015) with ‘‐‐stranded=reverse‐a 10‐t CDS‐m intersection‐nonempty’ options (Tables S1 and S2). The functional annotation of each gene was obtained by the Bacterial and Viral Bioinformatics Resource Center (BV‐BRC) platform (Olson et al. 2023).

### 2.6 Gene Expression Analysis

To explore similarities among RNAseq samples, non‐metric multidimensional scaling (NMDS) ordination was performed on VST‐transformed data. Environmental fitting (ENVFIT) analysis with 999 permutations was then applied to test whether environmental variables (temperature and salinity) significantly correlated with the NMDS ordination axes. We used DESeq2 to find differentially expressed genes across the experimental conditions (Love et al. 2014). The DESeq2 normalised counts were used as an input for *softclustering* analysis (Schwämmle and Jensen 2010). A matrix of *n* genes per six conditions (three salinities at two temperatures) was employed as input data. Softclustering assigns genes with similar responses to salinity and temperature conditions (such as upward or downward trends) to the same cluster (Tables S3 and S4). The ClusterJudge method was utilised to choose the number of gene clusters (Pasculescu 2023), and the Fuzzy c‐means algorithm was used to standardise the data within clusters. The optimal value of the *m* parameter in the Mfuzz algorithm was estimated through randomisation (Schwämmle and Jensen 2010). For annotating gene functional categories, the protein sequences of RS9907 and WH5701 genomes were compared against the Cluster of Orthologous Groups categories (COG) database using BLASTp, retrieving best hits with an *e*‐value threshold of 0.01. To obtain the set of differentially expressed genes across the salinity range under optimal and low temperatures, we conducted the likelihood ratio test (LRT) within DESeq2 with default settings and a *p*‐value cutoff of 0.01.

## 3 Results

### 3.1 Physiological Response of Model Marine and Euryhaline *Synechococcus* Strains to Salinity Acclimation Under Optimal and Cold Temperature Conditions

**FIGURE 1:**
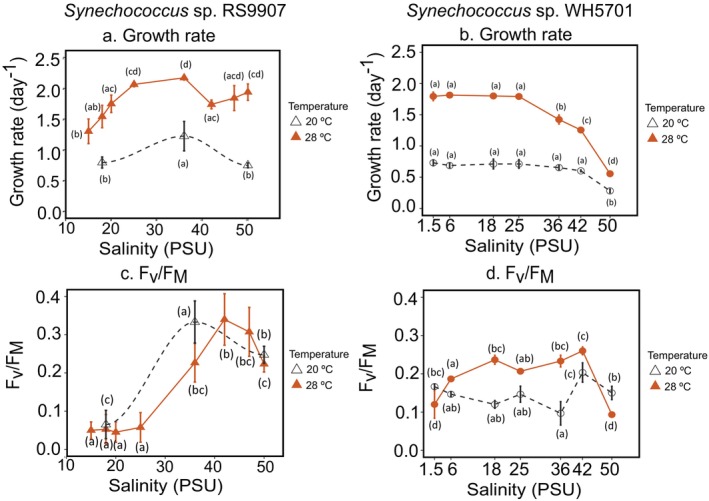
Growth rates (a, b) and maximum quantum yield of the photosystem II (*F*_v_/*F*_M_, c, d) along the salinity acclimation of *Synechococcus* sp. RS9907 (left panels) and *Synechococcus* sp. WH5701 (right panels). At each salinity condition, the average and standard deviation of three or four biological replicates are shown. Closed symbols indicate 28°C and 30°C for RS9907 and WH5701, respectively, while open symbols indicate 20°C and 15°C. Lowercase letters denote statistically significant differences between salinities for each strain and temperature condition (ANOVA analysis, *p*‐value < 0.05 and Tukey's range test). When comparing values between temperatures for each parameter and strain, differences were significant in all cases (Student's *t* test, *p*‐value < 0.05), except for *F*_V_/*F*_M_ values in strain RS9907 (c) and 1.5 PSU condition in strain WH5701 (d).

The salt acclimation experiments with the marine strain RS9907 were conducted at two temperature conditions: 20°C, which is near its cold threshold, and 28°C, which is close to its optimal growth temperature, as reported earlier (Escribano‐Gómez et al. 2025; Labban et al. 2021; Palacio et al. 2020). At 28°C, RS9907 was able to grow between 15 and 50 PSU under our experimental conditions (Figures 1a and S2b). Maximum growth was observed at 36 PSU (2.16 day^−1^). Above and below 36 PSU, growth decreased significantly (ANOVA; *p*‐value < 0.05), down to 1.17 day^−1^ at 15 PSU and 1.93 day^−1^ at 50 PSU. At 20°C, growth experiments were conducted at the contrasting salinities 18, 36 and 50 PSU. A similar growth pattern was obtained along the salinity gradient, with maximum rates at 36 PSU (1.22 day^−1^, Figures 1a and S2a). Overall, growth rates of RS9907 at 20°C decreased significantly as compared to 28°C (Student's *t* test, *p*‐value < 0.05, Figures 1a and S2).

In the case of the euryhaline WH5701, salt acclimation experiments were conducted at its optimum growth temperature, 30°C, and at 15°C, which is close to its cold threshold (see Section 2). At 30°C, growth rates of WH5701 remained relatively high (ca. 1.8 day^−1^) over a wide range of salinities from 1.5 to 25 PSU and decreased thereafter down to 50 PSU (0.55 day^−1^, ANOVA, *p*‐value < 0.05). Under the cold condition 15°C, growth rates remained close to 0.7 day^−1^ across a wide range of salinities and decreased significantly at 50 PSU (0.28 day^−1^, ANOVA, *p*‐value < 0.05). As in RS9907, growth rates of WH5701 at 15°C were significantly lower than those at 30°C along the whole salinity gradient (Student's *t* test, *p*‐value < 0.05; Figure 1b).

### 3.2 Photosynthetic Performance in Salt Acclimated Samples of RS9907 and WH5701 Strains

To investigate the photochemical performance of both strains along the salinity gradient, we measured the maximal quantum yield of the PSII (*F*_V_/*F*_M_). In RS9907, the *F*_v_/*F*_M_ yield was consistently low from 15 to 25 PSU (below 0.1) at optimal growth temperature, despite growth rates increasing 1.5‐fold over this range. At 36 PSU and above, the *F*_V_/*F*_M_ increased beyond 0.2 and remained high at 50 PSU. Notably, there were no significant differences in the *F*_V_/*F*_M_ values at both temperatures tested for RS9907 (20°C and 28°C, Figure 1c). In the case of WH5701, the *F*_V_/*F*_M_ yield followed a unimodal response from 6 to 42 PSU (with *F*_V_/*F*_M_ values above 0.2), and minimum values were found at the low and high salinity conditions, 1.5 and 50 PSU. Under cold temperature conditions (15°C), the *F*_V_/*F*_M_ yields were significantly lower as compared to 30°C (Student's *t* test, *p*‐value < 0.05; Figure 1d), specifically between the range 18 to 36 PSU (Figure 1d). However, similar *F*_V_/*F*_M_ yields were obtained at both temperatures in the salinity limits (1.5–6 PSU and 42–50 PSU).

**FIGURE 2:**
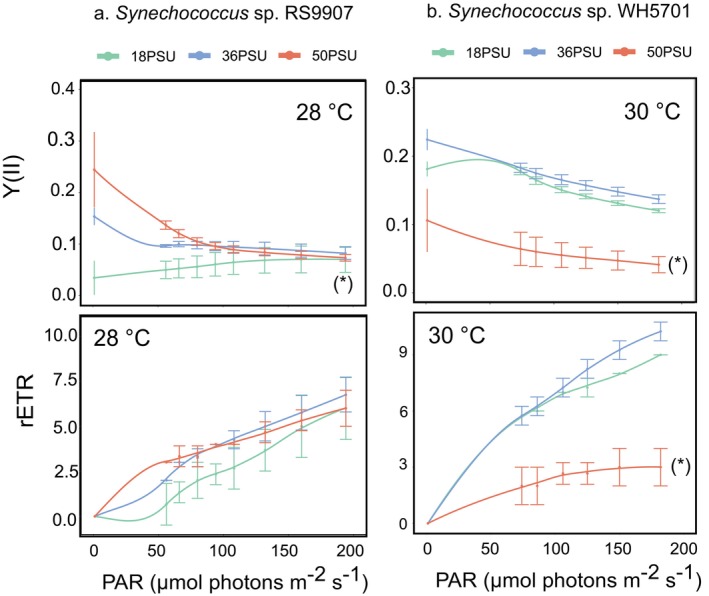
Photochemical parameters under different salinity conditions of strains *Synechococcus* sp. RS9907 (a) and *Synechococcus* sp. WH5701 (b). The effective quantum yield of PSII (*Y*(II)) and the relative electron transport rate (rETR) of salt acclimated cultures were plotted along an increasing gradient of photosynthetically active radiation (PAR). Green, blue and red colours refer to 18, 36 and 50 PSU. At each salinity condition, the average and standard deviation of three or four biological replicates are shown. Asterisks denote statistically significant differences in each salinity condition across light intensity (analysis of repeated‐measures ANOVA and pairwise *t* test; *p*‐value < 0.05).

The effective photochemical quantum yield of PSII *Y*(II) and the relative ETR (electron transport rate) of salt acclimated cultures were measured to gain further insights into the overall state of photosynthesis under three contrasting salinity conditions (18, 36 and 50 PSU). These parameters were only measured at the optimal growth temperature in both *Synechococcus* strains (28°C and 30°C, Figure 2) as we could not obtain entirely robust measurements under cold temperature conditions.

In RS9907, the *Y*(II) of cultures acclimated at 36 and 50 PSU decreased with the increase of PAR, which is indicative of a progressive closing of PSII reaction centres in response to light intensity (Campbell et al. 1998). However, cultures acclimated at 18 PSU showed an exceptionally low *Y*(II) yield, and only a minor response was measured with increasing light intensity (Figure 2a). This suggests that the PSII activity of RS9907 is impaired under low salt conditions. By contrast, the rETR parameter was not impacted by salinity in this strain, indicating that the photosynthetic electron transport remained functional, even under salinity stress.

In the case of WH5701, PSII reaction centres remained responsive to increasing light intensity under all salinity conditions, according to the *Y*(II). However, a lower *Y*(II) yield was obtained at 50 PSU as compared to 18 and 36 PSU. Similarly, a 65%–70% reduction of rETR was observed at 50 PSU compared to 36 or 18 PSU (Figure 2b, repeated‐measures ANOVA and pairwise *t* test; *p*‐value < 0.05). These results suggest a severe impact of the high salinity condition on the functionality of the photosynthetic apparatus in strain WH5701.

### 3.3 Analysis of Differential Gene Expression During the Salinity Acclimation Under Cold and Optimum Temperature Conditions in Strains RS9907 and WH5701

We analysed the global transcriptional response of both strains during the acclimation experiments by RNAseq. NMDS ordination revealed distinct clustering patterns among RNAseq samples in both strains (Figure S4). The ENVFIT analysis indicated that salinity significantly influenced the transcriptional ordination in RS9907 (*p* < 0.05), while for WH5701, both salinity and temperature were found to significantly influence the transcriptional ordination (*p* < 0.05). To identify significant differences in the gene expression profile across salinity conditions, we conducted a LRT test for each strain (*α* = 0.01; see Section 2). The LRT test identified 71% of WH5701 genes (2099 genes) differentially regulated by salinity, in contrast to only 6% of genes (188 genes) in RS9907 (Tables S5 and S6). If we differentiate between low and optimal temperature conditions, approximately half of WH5701 genes were differently regulated by salinity at each temperature condition. In the case of RS9907, 51 genes (2%) and 244 genes (8%) were differentially regulated by salinity at 20°C and 28°C, respectively (Tables S5 and S6).

**FIGURE 3:**
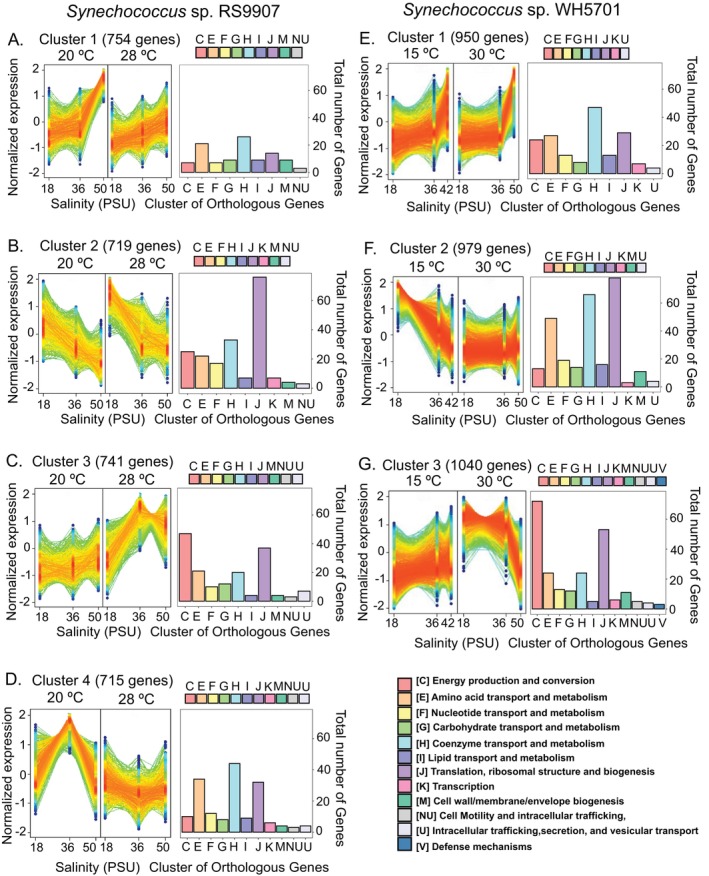
Identified gene clusters based on expression profiles across different salinity and temperature conditions and their corresponding annotation in functional COG categories of *Synechococcus* sp. RS9907 (A–D) and *Synechococcus* sp. WH5701 (E–G). For each cluster, the normalised expression values are shown in the left panel and the main functional Cluster Orthologous Gene (COG) categories in the right panel. The number of genes assigned to COG categories was low; the COG categories displayed contained at least three genes. The colours of the dots and lines indicate the membership value assigned by the fuzzy c‐means soft clustering of each gene. The values range from 1 (red, indicating a high score) to 0.5 (blue, indicating a low score). The total number of genes assigned to each cluster is shown.

We performed a *clustering* analysis to identify genes with a similar expression response across salinity and temperature conditions, based on DESeq2 normalised counts as input data (see Section 2, Tables S3 and S4). Genes in each cluster were annotated against the Cluster of Orthologous Groups database, to identify the main COGs functional categories represented in each cluster (Figure 3). In RS9907, four clusters were identified considering the expression profile of each gene along the salinity range and under both temperature conditions (Figures 3A–D and S5a). In the euryhaline strain, WH5701, only three clusters were found (Figures 3E–G and S5b). Some of these clusters showed similarities in their expression profile along the salinity gradient and COG functional categories distribution, so their results are jointly explained below.

**FIGURE 4:**
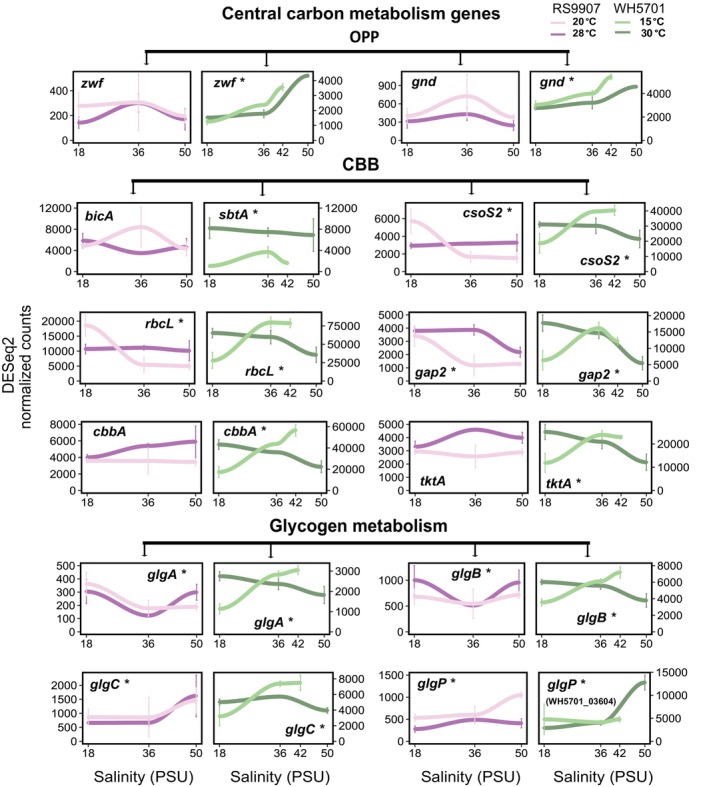
Gene expression values of a selection of individual genes related to central carbon metabolism in both *Synechococcus* strains along the salinity gradient and at two growth temperature conditions. CBB, Calvin Benson–Bassham cycle; OPP, oxidative pentose phosphate pathway. Asterisks denote differential expression along the salinity gradient (LTR test, *ɑ* = 0.01). Light colours refer to low temperatures (20°C and 15°C for RS9907 and WH5701, respectively) and dark colours refer to optimal growth temperatures (28°C and 30°C for RS9907 and WH5701, respectively). RS9907 values appear in pink and WH5701 values appear in green.

**FIGURE 5:**
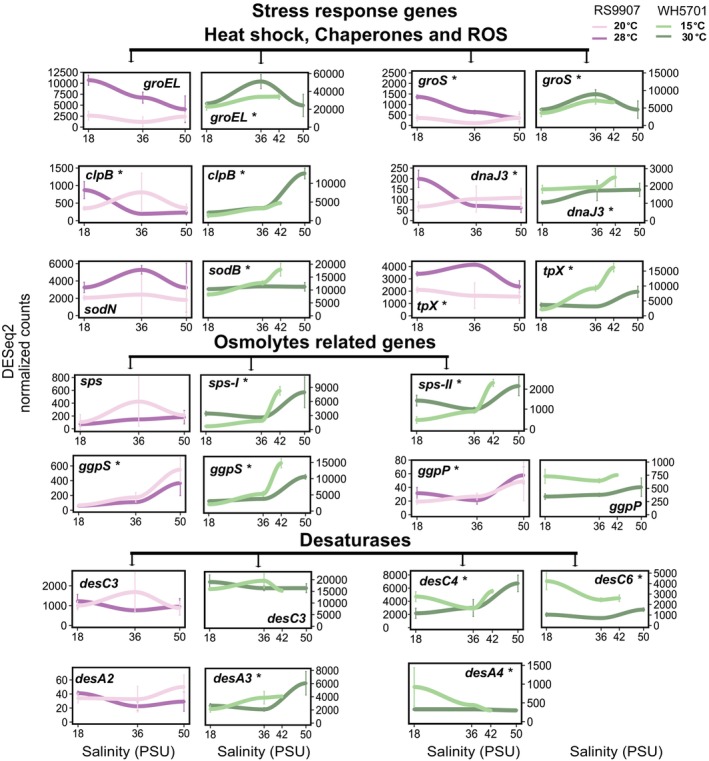
Gene expression values of a selection of individual genes related to stress response in both *Synechococcus* strains along the salinity gradient and at two growth temperatures conditions. At each salinity, the average of three biological replicates is shown, and error bars represent the standard deviation. ROS, reactive oxidative species. Asterisks denote differential expression along the salinity gradient (LTR test, *ɑ* = 0.01). Light colours refer to low temperatures (20°C and 15°C for RS9907 and WH5701, respectively) and dark colours refer to optimal growth temperatures (28°C and 30°C for RS9907 and WH5701, respectively). RS9907 values appear in pink and WH5701 values appear in green.

Genes in cluster 1 of RS9907 or WH5701 were transcriptionally induced at high salinity (50 PSU, Figure 3E). In the case of RS9907, the upregulation at 50 PSU was enhanced under cold conditions (20°C; Figure 3A). A relatively low fraction of genes could be assigned to COG functional categories in this cluster (105 of 754 genes in RS9907 and 172 of 950 genes in WH5701), with H (coenzyme transport and metabolism) and E (amino acid transport and metabolism) being the most prevalent. Some relevant functional genes were included in cluster 1, such as *glgP*, involved in glycogen degradation (Figure 4), and genes related to compatible osmolyte synthesis (*ggpS*, *ggpP*; Figure 5). While the upregulation of osmolyte synthesis genes at 50 PSU was observed regardless of the temperature condition (i.e., *sps‐I‐II* in WH5701, *ggpS* in both strains, Figure 5), *glgP* was upregulated at 50 PSU only under cold conditions in RS9907 (Figure 4). Thus, this suggests an interaction of both stressors in their regulation. As a major difference in the transcriptional response of both strains, some carbon metabolism genes, such as those involved in the pentose phosphate pathway (*zwf*, *gnd*), showed maximum expression at 50 PSU in WH5701 but not in RS9907 (Figure 4). Genes related to bicarbonate transporters (*cmpABCD*) and some fatty acid desaturase genes (*desC4, desA3*), only detected in the WH5701 genome, were also identified within cluster 1, being upregulated at high salinity (Figures 5, S5 and S6).

**FIGURE 6:**
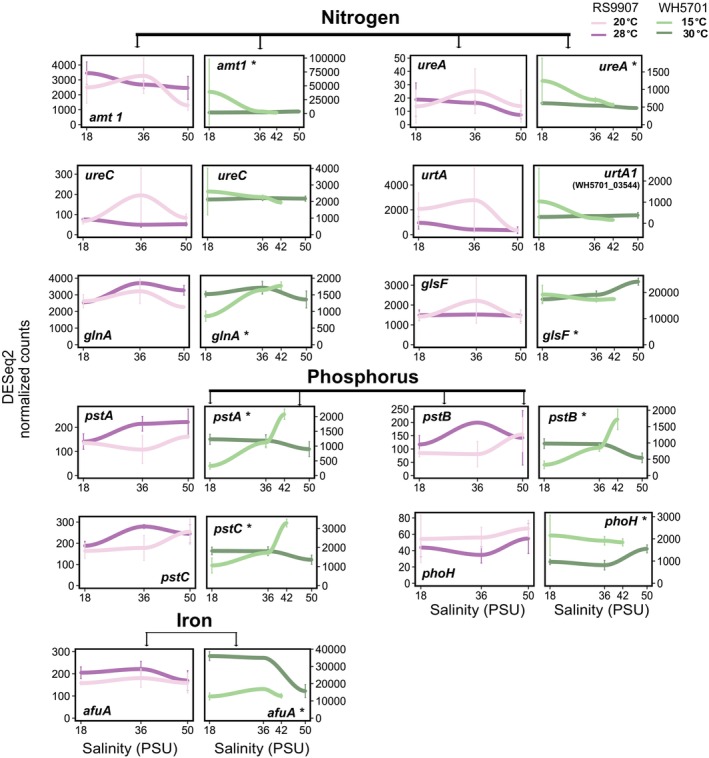
Gene expression values of a selection of individual genes related to nitrogen, phosphate and iron metabolism in both *Synechococcus* strains along the salinity gradient and at two growth temperature conditions. At each salinity, the average of three biological replicates is shown, and error bars represent the standard deviation. Asterisks denote differential expression along the salinity gradient (LTR test, *ɑ* = 0.01). Light colours refer to low temperatures (20°C and 15°C for RS9907 and WH5701, respectively) and dark colours refer to optimal growth temperatures (28°C and 30°C for RS9907 and WH5701, respectively). RS9907 values appear in pink and WH5701 values appear in green.

**FIGURE 7:**
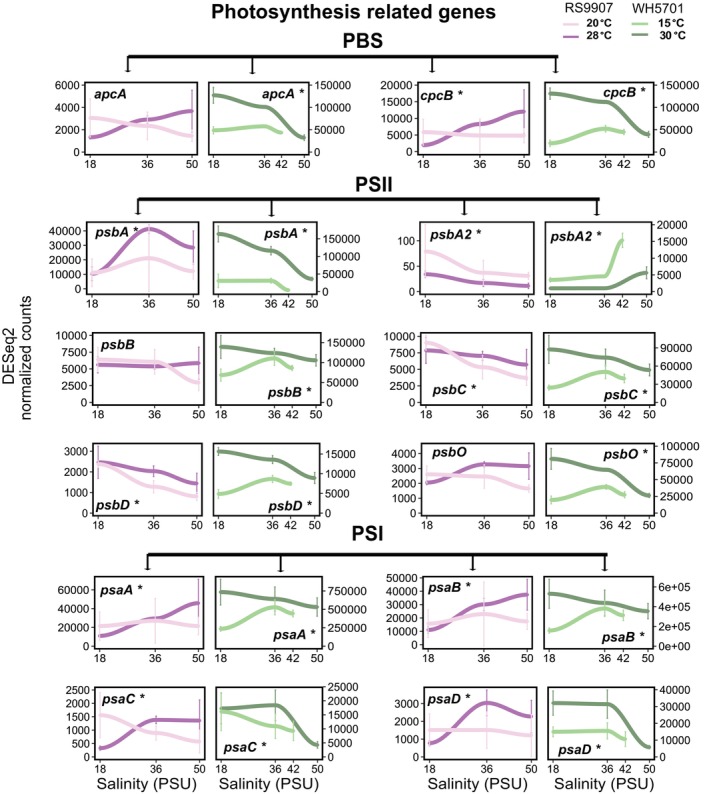
Gene expression values of a selection of photosynthesis related genes in both *Synechococcus* strains along the salinity gradient and at two growth temperature conditions. At each salinity, the average of three biological replicates is shown, and error bars represent the standard deviation. PBS, phycobilisomes; PSI, photosystem I; PSII, photosystem II. Asterisks denote differential expression along the salinity gradient (LTR test, *ɑ* = 0.01). Light colours refer to low temperatures (20°C and 15°C for RS9907 and WH5701, respectively) and dark colours refer to optimal growth temperatures (28°C and 30°C for RS9907 and WH5701, respectively). RS9907 values appear in pink and WH5701 values appear in green.

The expression of genes in cluster 2 generally showed a negative trend with salinity (Figure 3). The downregulation of genes at 50 PSU was observed either under optimal or low temperature conditions, or in some cases, under both temperatures in RS9907. Genes related to the general stress response (heat shock proteins and chaperones, *groEL*, *groL2S* and *clpP*; Figures 5 and S6) and nitrogen assimilation gene (*amt1*, Figure 6) were upregulated at 18 PSU under optimal growth temperature in RS9907 (28°C). By contrast, carbon fixation genes (RuBisCO, *rbcLS*, *csoS2*; Figure 4) were upregulated at low salinity but only under low temperature in this strain (i.e., 18 PSU and 20°C), while PSII subunits genes (*psbA2CD*) were upregulated at 18 PSU at both temperatures (Figure 7). In the case of WH5701, genes in cluster 2 also showed a negative trend with salinity, but only under low temperature, 15°C (Figure 3F). Within this cluster, genes involved in nitrogen assimilation (*amt1, urtA1* and *ureC*) were upregulated under conditions of low salinity and temperature (Figure 6). Some of the genes uniquely assigned to cluster 2 in WH5701 strain (but not in cluster 2 of RS9907) were related to amino acid biosynthesis (*argDG, metK* and *trpB*; Figure S6) and DNA damage repair (*recA*, *uvrA* and *lexA*). The fatty acid desaturases genes *desC6* and *desA4*, only identified in WH5701 genome, also displayed a negative trend with salinity and temperature, with maximum expression at 18 PSU and 15°C (Figure 5). This highlights a different regulation pattern as compared to *desC4* and *desA3* gene variants (see above).

In cluster 3, most genes were functionally annotated to COG category C (energy production and conversion) and followed an expression profile that paralleled growth rates under optimal temperature conditions (Figure 3). In the case of RS9907, gene expression was minimal at 18 PSU and increased with salinity under optimal growth temperature (Figure 3C), while for WH5701, maximal expression was found at 18 PSU and minimal at 50 PSU under optimum growth conditions (30°C; Figure 3G). Some of the genes in cluster 3 were related to phycobilisomes (*apcAEF*, *cpcAB*), PSII (*psbAO*) and PSI (*psaABD*) subunits genes in both strains (Figure 5). However, genes related to carbon fixation and metabolism (*csoS2, rbcLS*) were assigned to cluster 3 of WH5701 but not in RS9907. Notably, while the expression of the latter genes followed the growth rate pattern at 30°C in WH5701, at low temperature, these genes showed maximum expression under salinity stress conditions (42 PSU). Genes associated with some membrane transporters (*aqpZ, mntABCD*; Figure S6), only detected in WH5701 genome, were also assigned to cluster 3 of WH5701.

Finally, the transcriptional response of RS9907 genes in cluster 4 was unimodal at 20°C, whereas at 28°C, gene expression decreased with high salinity, reaching a minimum level at 50 PSU (Figure 3D). In this cluster, most of the identified genes were related to coenzyme and amino acids transport and metabolism (COG H and E; Figure 3D), including genes involved in nitrogen assimilation (*ureC, urtA* and *glsF*; Figure 6). Furthermore, genes associated with translation, ribosomal structure and biogenesis (COG J), such as ribosomal subunits, were identified. Genes involved in stress response, including the fatty acid desaturase *desC3* and some chaperones (*clpBC*), also followed this expression pattern (Figure S6).

## 4 Discussion

Changes in global salinity of the oceans are indicative of a freshening trend occurring in high latitudes, while predictions indicate that surface tropical waters will become saltier (Cheng et al. 2020; Durack and Wijffels 2010; Pontes and Menviel 2024; Sathyanarayanan et al. 2021). However, very few studies have addressed the impact of salinity on marine *Synechococcus*, and particularly in combination with temperature, which is also changing at the global scale (Garcia‐Soto et al. 2021). By studying the transcriptional responses that underpin temperature and salinity acclimation in different *Synechococcus* clades, we can gain a better understanding of how their physiology adjusts to changes in thermohaline conditions.

The salinity tolerance range of WH5701 is in agreement with previous studies on other euryhaline *Synechococcus* strains (Marsan Wilfred 2016; Xia et al. 2015, 2020). By contrast, RS9907 showed a more limited tolerance to low salinity as compared to other marine strains from subcluster 5.1, which typically grow down to 15 PSU (Marsan Wilfred 2016; Xia et al. 2015, 2020). However, it is worth noticing that RS9907 maintained growth rates above 1 day^−1^ at salinity values down to 18 PSU. This suggests that marine *Synechococcus* strains are rather resilient to changes in oceanic freshening events, which typically produce only mild modifications of salinity conditions in surface waters (Sathyanarayanan et al. 2021).

At the physiological level, we found evidence of a decreased capacity to perform photosynthesis at salinity stress conditions in both strains (18 PSU in RS9907 and 50 PSU in WH5701), likely explaining the drop in their growth rates. Similarly, a previous study on the marine cyanobacterium *Prochlorococcus* demonstrated that their photosynthetic performance was reduced at relatively low salinity concentration (24–27 PSU), while in the freshwater *Synechocystis* sp. PCC6803, the drop in *F*_V_/*F*_M_ was observed under relatively high salinity (≈29 PSU; Díaz‐Troya et al. 2020; He et al. 2022). Based on our photophysiological measurements on strain RS9907, even if the PSII activity at 18 PSU remained minimal, growth rates were sustained. We hypothesise that RS9907 may activate cycling electron flow around PSI under low salinity conditions as a protective mechanism to prevent the overreduction of electron carriers and adjust the balance between ATP and NADPH (Jeanjean et al. 1998; Stirbet et al. 2018). It is remarkable that a *Synechocystis* mutant strain lacking PSI exhibited a substantial impairment in salt tolerance, suggesting that cyclic electron flow around PSI was imperative for maintaining tolerance to salt stress (Howitt et al. 2001). On the other hand, the decreased rETR of WH5701 at 50 PSU indicates that salt stress inhibited the electron transfer between their PSII and PSI reaction centres in the latter strain, as has been previously observed in other cyanobacteria (Hu et al. 2014; Lu and Zhang 1999; Qiu et al. 2022; Swapnil et al. 2015). Overall, these results highlight a less robust photosynthetic stress response in the euryhaline *Synechococcus* strain as compared to its marine counterpart.

In terms of temperature, a decline in *F*_V_/*F*_M_ is generally observed in response to cold conditions (9°C–18°C) in marine cyanobacteria (Guyet et al. 2020; Mackey et al. 2013; Pittera et al. 2014; Six et al. 2021; Varkey et al. 2016). This response was clearly observed in the case of WH5701 (from 30°C to 15°C), but not in RS9907, which showed *F*_V_/*F*_M_ similar values under optimal (28°C) and low temperature (20°C) across the salinity range. This different response may be partly attributed to the fact that the cold‐acclimation temperature was lower in strain WH5701 than in RS9907. However, differences in pigment composition may also underlie the observed differences. While RS9907 is a phycoerythrin‐type strain, WH5701 contains only phycocyanin in their phycobilisomes (Six et al. 2007). It has been demonstrated that strains with a higher proportion of phycoerythrin exhibit stronger state transitions (Mackey et al. 2013), which protect the photosynthetic apparatus. Furthermore, temperature also influences the thermostability of phycobilisomes, with phycocyanin being the least stable of all pigment types (Pittera et al. 2017).

Despite the apparent photosynthetic resilience of RS9907 to stress conditions, this strain demonstrated a reduced ability to modulate gene expression in response to salinity, with only 6% of genes differentially expressed. This suggests a less flexible transcriptional acclimation strategy compared to the euryhaline strain, which typically thrives in environments with fluctuating salinity conditions. It should be noted that the acclimation procedure was longer for RS9907 (ca. 6 months) than for WH5701 (ca. 3 weeks), which may partly contribute to explain this result. However, previous results in freshwater strains such as *Synechocystis* sp. PCC6803 showed that most of the variations in gene expression were transient following 24 h of salt exposure, and only 39 genes remained regulated after 5 days of incubation at 684 mM of NaCl (≈40 PSU; Marin et al. 2004). Thus, the fact that we observed such a large remodelling of the WH5701 transcriptome under different salinity conditions after at least 16 generations suggests that this response is not transient. This agrees with previous results in the euryhaline strain *Synechococcus* sp. HK05 (Xia et al. 2023), potentially contributing to their capacity to acclimate to a broad range of salinities.

A core set of genes involved in glycogen degradation and osmolyte synthesis was consistently upregulated under high salinity conditions in both strains, likely reflecting an increased energetic demand and the need for osmotic adjustment. Yet, the response of specific osmolytes seemed to vary between both strains. In RS9907, *ggpS* and *ggpP* genes involved in glucosylglycerol accumulation were upregulated at 50 PSU, but no induction of sucrose transcript accumulation was observed, as previously found in *Synechococcus* sp. WH8102 (Lu et al. 2006). Sucrose can be only transiently accumulated in response to salinity in some strains (Kirsch et al. 2019; Klähn et al. 2021; Marin et al. 2004; Warr et al. 1985), and thus, we may have missed this specific response after the long salinity acclimation period in RS9907 (Kirsch et al. 2019; Klähn et al. 2021; Marin et al. 2004). However, sucrose synthesis genes were consistently upregulated under warm conditions in a previous study where RS9907 was long‐term acclimated to a temperature gradient from 20°C to 33°C (Escribano‐Gómez et al. 2025), or in the freshwater strain *Synechocystis* sp. PCC6714 (Warr et al. 1985). We hypothesise that sucrose synthesis may be a specific response to changes in temperature in RS9907, rather than salinity conditions, with the latter potentially requiring accumulation of glucosylglycerol. In strain WH5701, under high salinity, the expression level of glucosylglycerol synthesis genes was higher than those of sucrose, as previously found for other euryhaline *Synechococcus* strains (Ludwig and Bryant 2012; Xia et al. 2020), indicating that WH5701 primarily accumulates glucosylglycerol rather than sucrose (Kirsch et al. 2019; Klähn et al. 2021; Marin et al. 2004; Xia et al. 2023).

In both WH5701 and RS9907, the expression of genes involved in photosynthesis and central carbon metabolism was differently regulated by the combined effect of salinity and temperature. Notably, the expression of a substantial fraction of photosynthetic genes (e.g., *psbA*, *psbO*, *psaA*, *psaB*, *psaC* and *psaD*) mirrored the growth profile in both strains. In euryhaline and freshwater *Synechococcus* strains or in *Prochlorococcus marinus* AS9601, transcript levels of PSI were downregulated at high salinity, likely reflecting inhibitory effects on the electron‐transfer activity of PSI (Al‐Hosani et al. 2015; Allakhverdiev et al. 2000; Kanesaki et al. 2002; Ludwig and Bryant 2012). Conversely, under low salt conditions, the expression of photosynthetic genes was upregulated in *P. marinus* NATL1A, but downregulated in *P. marinus* MED4, indicating heterogeneity in photosynthetic gene expression in response to low salinity stress (He et al. 2022).

In the case of carbon fixation genes, the impact of salt conditions also differs between cyanobacterial isolates (Al‐Hosani et al. 2015; Billis et al. 2014; Xia et al. 2023). Yet, notably, salt stress and low temperature induced the upregulation of carbon fixation genes, in a seemingly common response in both strains analysed here. We hypothesise that the combined impact of both stressors induces a significant drop in CO_2_ fixation rates, thereby resulting in the production of reactive oxidative species (ROS), which can lead to photosystem damage (Murata et al. 2007; Yang et al. 2020). Thus, upregulating carbon fixation genes may be a compensatory mechanism to minimise these detrimental effects. A substantial amount of membrane transporter genes was induced during the salinity acclimation in WH5701, a response that was not found in RS9907. Similarly, an upregulation of membrane transporter genes was also found in the euryhaline strain *Synechococcus* sp. HK05 and in the proteome of *Synechocystis* in response to salt stress (Huang et al. 2006; Xia et al. 2023).

Finally, the expression of fatty acid desaturase genes was not significantly regulated in response to temperature or salinity in RS9907 after the long acclimations, likely because changes in the expression of these genes are typically transient (Ludwig and Bryant 2012; Mironov et al. 2012). However, in the case of *Prochlorococcus*, long acclimations to salinity or temperature stress conditions induced changes in the expression of fatty acid desaturases (Alonso‐Sáez et al. 2023; He et al. 2022), as we observed here for strain WH5701. Notably, the *desC4* and *desA3* genes, typically associated with cold‐adapted *Synechococcus* genomes (Breton et al. 2020), were upregulated under high salinity conditions (50 PSU) in WH5701. In contrast, the *desA2* variant, mostly found in warm thermotypes (Pittera et al. 2018), was not regulated by salinity, while *desC6* and *desA4* genes were upregulated under low salinity and cold conditions. This suggests selective expression of fatty acid desaturase variants to modulate membrane fluidity in response to environmental stress conditions. The insertion or deletion of fatty acid desaturase genes in freshwater *Synechococcus* strains has been found to influence salt tolerance of their photosynthetic apparatus (Allakhverdiev et al. 1999, 2001). In future research, a comparable genetic modification analysis with marine *Synechococcus* strains would be crucial to elucidate the role of specific fatty acid desaturases in their thermohaline adaptation.

## 5 Conclusion

Our study highlights distinct physiological and transcriptional responses to salinity and temperature stress in marine and euryhaline *Synechococcus* strains, underscoring their divergent strategies for coping with environmental challenges. The euryhaline strain WH5701 exhibited a more extensive transcriptional remodelling to cope with changes in salinity compared to the marine strain RS9907, by inducing the expression of membrane transporters and carbon metabolism genes, among others. This versatile transcriptional response is likely crucial for survival in highly dynamic environments where rapid and substantial changes in salinity are common. Despite its stronger regulatory capacity, the euryhaline strain exhibited a less resilient photosynthetic activity under salinity stress conditions as compared to the marine strain, likely due to limitations imposed by its photosynthetic apparatus. A core of genes related to compatible osmolyte synthesis, glycogen degradation and photosynthesis were regulated by salinity in both strains, indicating some general mechanisms of acclimation in *Synechococcus*. Our work also reveals that the combined stress induced by temperature and salinity activates specific responses, such as the upregulation of carbon fixation genes and specific fatty acid desaturases (e.g., *desC6* and *desA4*). A deeper understanding of these responses will be crucial to uncertain molecular mechanisms of adaptation of *Synechococcus* to varying thermohaline conditions.

## Author Contributions

Conceptualization: L.A.‐S, Á.L.‐U., I.E.‐G. Data curation: I.E.‐G., R.P., R.L., L.A.‐S., Á.L.‐U. Formal analysis: I.E.‐G, R.P., R.L. Funding acquisition: L.A.‐S, Á.L.‐U. Investigation: I.E.‐G., R.P., R.L., U.A., M.V.‐L., L.A.‐S., Á.L.‐U. Formal analysis: I.E.‐G, R.P., R.L. Project administration: L.A.‐S. Visualisation: I.E.‐G. Writing – original draft preparation: I.E.‐G. Writing – reviewing and editing: L.A.‐S., R.P., R.L., Á.L.‐U.

## Funding

This work was supported by the Spanish Ministry of Economy and Competitiveness (RYC‐2012‐11404), Spanish Ministry of Science, Innovation and Universities (RTI2018‐100690‐BI00, PRE2019‐091180), Eusko Jaurlaritza, BIOMATRIX.

## Ethics Statement

The authors have nothing to report.

## Conflicts of Interest

The authors declare no conflicts of interest.

## Supporting information


**Figure S1:** Schematic diagram of the experimental approach and sample collection during the temperature and salinity acclimation of *Synechococcus* sp. RS9907 and *Synechococcus* sp. WH5701. The asterisks indicate samples collected for RNA extraction and transcriptome sequencing.
**Figure S2:** Growth rates at 20°C (a) and 28°C (b) along the salinity acclimation process of *Synechococcus* sp. RS9907. At each salinity condition, the average and standard deviation of three or four biological replicates is shown. Lowercase letters denote statistically significant differences between temperatures (analysis of variance [ANOVA]; *p*‐value < 0.05 and Tukey's range test).
**Figure S3:** Growth rates (a) and quantum yield of the photosystem II reaction centre (b) during the thermal acclimation of *Synechococcus* sp. WH5701. At each temperature condition, the average and standard deviation of three biological replicates is shown. Lowercase letters denote statistically significant differences between temperatures (analysis of variance [ANOVA]; *p*‐value < 0.05 and Tukey's range test).
**Figure S4:** Non multidimensional scaling plot of RNA samples obtained during the acclimation to salinity and temperature of *Synechococcus* sp. RS9907 (A) and WH5701 (B). Green, light blue, dark blue and red colours refer to salinity samples, 18, 36, 42 and 50 PSU, respectively. Circle and triangle shapes indicate the temperature of acclimation in both strains. The ENVFIT analysis indicates that salinity significantly influences the transcriptional ordination in RS9907, while in WH5701, both salinity and temperature influence the transcriptional ordination (*p* < 0.05).
**Figure S5:** Selection of functional genes identified in each of the clusters based on expression profiles across different salinity and temperature conditions of *Synechococcus* sp. RS9907 (a) and *Synechococcus* sp. WH5701 (b). For each cluster, the normalised expression values are shown, within right and left panels indicate both temperatures of acclimation, 20°C and 28°C in RS9907 and 15°C and 30°C in WH5701, respectively. The colours of the dots and lines indicate the membership value assigned by the fuzzy c‐means soft clustering of each gene. The values range from 1 (red, indicating a high score) to 0.5 (blue, indicating a low score). The total number of genes assigned to each cluster is shown. TCA refers to tricarboxylic acid cycle genes and OPP indicate oxidative pentose phosphate pathway genes.
**Figure S6:** Gene expression values of a selection of individual genes in both *Synechococcus* strains along the salinity gradient and at two growth temperatures conditions. (a) Membrane transporters genes only identified in WH5701 genome, (b) Genes involved in amino acid metabolism, (c) Genes related to stress response. At each salinity, the average of three biological replicates is shown, and error bars represent the standard deviation. Asterisks denote differential expression along the salinity gradient (LTR test, *ɑ* = 0.01). Light colours refer to low temperatures (20°C and 15°C for RS9907 and WH5701, respectively) and dark colours refer to optimal growth temperatures (28°C and 30°C for RS9907 and WH5701, respectively). RS9907 values appear in pink and WH5701 values appear in green.
Supporting data available at Zenodo (10.5281/zenodo.17608429).

**Table S1:** Read counts obtained by HTSeq for each protein‐coding gene in *Synechococcus* sp. RS9907 in the samples collected during the salinity range. The sample names include the temperature, salinity and the replicate number. Gene IDs, annotated gene products and Refseq Locus Tag according to the Bacterial and Viral Bioinformatics Resource Center (BV‐BRC) are shown.

**Table S2:** Read counts of *Synechococcus* sp. WH5701 obtained by HTSeq for each protein‐coding gene during the salinity gradient. The sample names comprise the temperature, salinity and the replicate number. The gene IDs, the annotated gene products and the RefSeq Locus Tag are shown according to the Bacterial and Viral Bioinformatics Resource Center (BV‐BRC).

**Table S3:** Softcluster membership, probability scores and functional annotation against the Cluster of Orthologous Group (COG) database for each RS9907 protein‐coding gene. Gene IDs according to the Bacterial and Viral Bioinformatics Resource Center (BV‐BRC) are shown.

**Table S4:** Softcluster membership, probability scores and functional annotation against the Cluster of Orthologous Groups categories (COG) database for each WH5701 protein‐coding gene. Gene IDs according to the Bacterial and Viral Bioinformatics Resource Center (BV‐BRC) are shown.

**Table S5:** Likelihood ratio test (LRT) results of gene expression in WH5701 without considering temperature effects (a), at optimal temperature (b) and at low temperature (c). Gene IDs according to the Bacterial and Viral Bioinformatics Resource Center (BV‐BRC) are shown. The genes listed are significantly regulated by salinity according to LRT results (*p*‐adj < 0.01).

**Table S6:** Likelihood ratio test (LRT) results of gene expression analysis in RS9907 without considering temperature effect (a), at optimal temperature (b) and at low temperature (c). Gene IDs according to the Bacterial and Viral Bioinformatics Resource Center (BV‐BRC) are shown. The genes listed are significantly regulated by salinity according to LRT results (*p*‐adj < 0.01).

## Data Availability

The authors confirm that the data supporting the findings of this study are available within the article, Supporting Information and in the open‐access repository Zenodo (10.5281/zenodo.17608429). The sequence dataset has been deposited in the European Nucleotide Archive (ENA) repository under project PRJEB89907 (accession numbers ERS24827365–ERS24827513).
