# Supplementary material for: Synechococcus Under Stress: Contrasting Physiological and Transcriptional Responses to Salinity and Temperature in Marine Versus Euryhaline Strains
Source: Environ Microbiol Rep. 2026 Jan 26;18(1):e70273. doi: 10.1111/1758-2229.70273 (PMC12834511; doi:10.1111/1758-2229.70273)
Supplement: Supplementary file 1 — Figure S1: Schematic diagram of the experimental approach and sample collection during the temperature and salinity acclimation of Synechococcus sp. RS9907 and Synechococcus sp. WH5701. The asterisks indicate samples collected for RNA extraction and transcriptome sequencing. Figure S2: Growth rates at 20°C (a) and 28°C (b) along the salinity acclimation process of Synechococcus sp. RS9907. At each salinity condition, the average and standard deviation of three or four biological replicates is shown. Lowercase letters denote statistically significant differences between temperatures (analysis of variance [ANOVA]; p‐value < 0.05 and Tukey's range test). Figure S3: Growth rates (a) and quantum yield of the photosystem II reaction centre (b) during the thermal acclimation of Synechococcus sp. WH5701. At each temperature condition, the average and standard deviation of three biological replicates is shown. Lowercase letters denote statistically significant differences between temperatures (analysis of variance [ANOVA]; p‐value < 0.05 and Tukey's range test). Figure S4: Non multidimensional scaling plot of RNA samples obtained during the acclimation to salinity and temperature of Synechococcus sp. RS9907 (A) and WH5701 (B). Green, light blue, dark blue and red colours refer to salinity samples, 18, 36, 42 and 50 PSU, respectively. Circle and triangle shapes indicate the temperature of acclimation in both strains. The ENVFIT analysis indicates that salinity significantly influences the transcriptional ordination in RS9907, while in WH5701, both salinity and temperature influence the transcriptional ordination (p < 0.05). Figure S5: Selection of functional genes identified in each of the clusters based on expression profiles across different salinity and temperature conditions of Synechococcus sp. RS9907 (a) and Synechococcus sp. WH5701 (b). For each cluster, the normalised expression values are shown, within right and left panels indicate both temperatures of acclimation, [file EMI4-18-e70273-s003.pdf]

Supporting Information

Environmental Microbiology Reports

Synechococcus under stress: contrasting physiological and transcriptional responses to salinity and temperature in marine *versus* euryhaline strains

Isabel Escribano-Gómez* (1), Rebeca Perez (1), Uxue Arrizabalaga (1), Raquel Liébana (1), Miriam Vergara-Len (1), Ángel López-Urrutia (2) and Laura Alonso-Sáez*(1)

Supporting Tables are available at the open-access repository Zenodo (10.5281/zenodo.17608429)

Table S1. Read counts obtained by HTSeq for each protein-coding gene in Synechococcus sp. RS9907 in the samples collected during the salinity range. The sample names include the temperature, salinity, and the replicate number. Gene IDs, annotated gene products and Refseq Locus Tag according to the Bacterial and Viral Bioinformatics Resource Center (BV-BRC) are shown.

Table S2. Read counts of *Synechococcus* sp. WH5701 obtained by HTSeq for each protein-coding gene during the salinity gradient. The sample names comprise the temperature, salinity, and the replicate number. The gene IDs, the annotated gene products and the RefSeq Locus Tag are shown according to the Bacterial and Viral Bioinformatics Resource Center (BV-BRC).

Table S3. Softcluster membership, probability scores and functional annotation against the Cluster of Orthologous Group (COG) database for each RS9907 protein-coding gene. Gene IDs according to the Bacterial and Viral Bioinformatics Resource Center (BV-BRC) are shown.

Table S4. Softcluster membership, probability scores and functional annotation against the Cluster of Orthologous Groups categories (COG) database for each WH5701 protein-coding gene. Gene IDs according to the Bacterial and Viral Bioinformatics Resource Center (BV-BRC) are shown.

Table S5. Likelihood Ratio Test (LRT) results of gene expression in WH5701 without considering temperature effects (a), at optimal temperature (b), and at low temperature (c). Gene IDs according to the Bacterial and Viral Bioinformatics Resource Center (BV-BRC) are shown. The genes listed are significantly regulated by salinity according to LRT results (p-adj < 0.01).

Table S6. Likelihood Ratio Test (LRT) results of gene expression analysis in RS9907 without considering temperature effect (a), at optimal temperature (b), and at low temperature (c). Gene IDs according to the Bacterial and Viral Bioinformatics Resource Center (BV-BRC) are shown. The genes listed are significantly regulated by salinity according to LRT results (p-adj < 0.01).

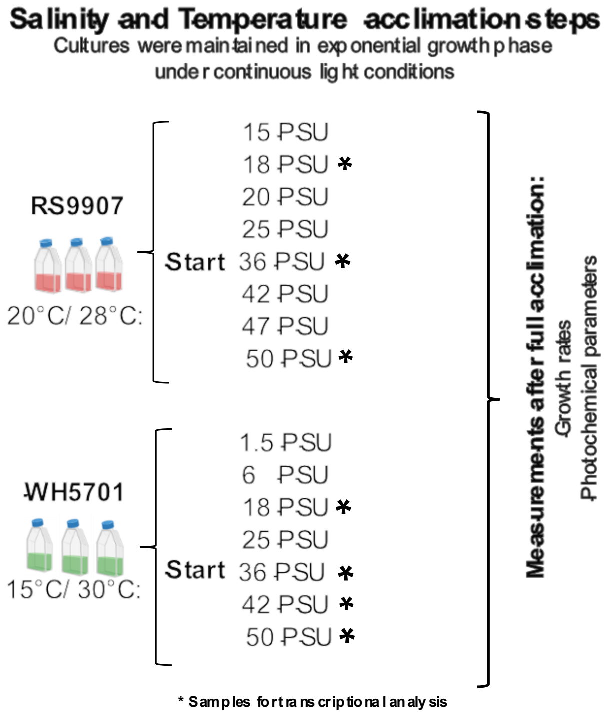

Figure S1. Schematic diagram of the experimental approach and sample collection during the temperature and salinity acclimation of *Synechococcus* sp. RS9907 and *Synechococcus* sp. WH5701. The asterisks indicate samples collected for RNA extraction and transcriptome sequencing.

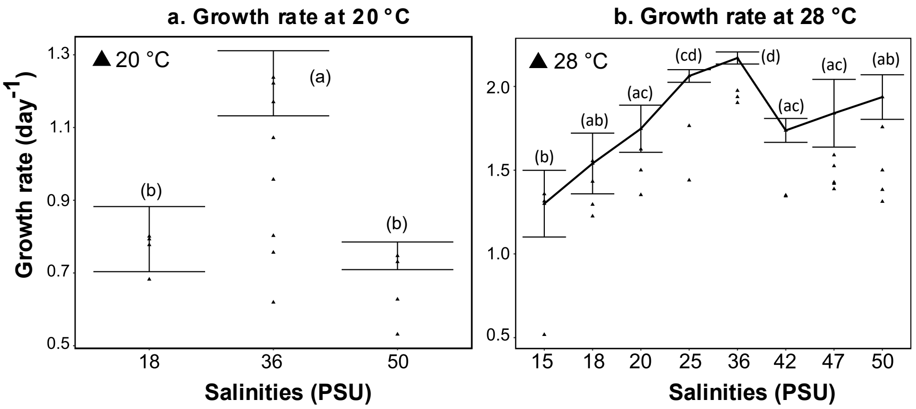

Figure S2. Growth rates at 20°C (a) and 28°C (b) along the salinity acclimation process of *Synechococcus* sp. RS9907. At each salinity condition, each dot represents the average of three or four biological replicates measured along the acclimation process. The error bars correspond to the standard deviation of the replicates corresponding to the last acclimation time point. Lowercase letters denote statistically significant differences between temperatures at the latter point (analysis of variance [ANOVA]; *p*-value <0.05 and Tukey's range test).

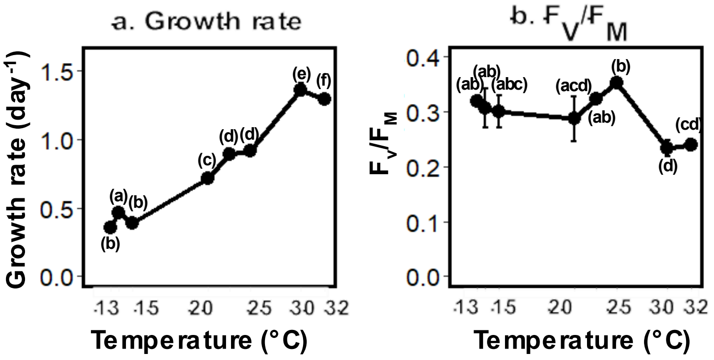

Figure S3. Growth rates (a) and quantum yield of the Photosystem II reaction center (b) during the thermal acclimation of *Synechococcus* sp. WH5701. At each temperature condition, the average and standard deviation of three biological replicates is shown. Lowercase letters denote statistically significant differences between temperatures (analysis of variance [ANOVA]; *p*-value <0.05 and Tukey's range test).

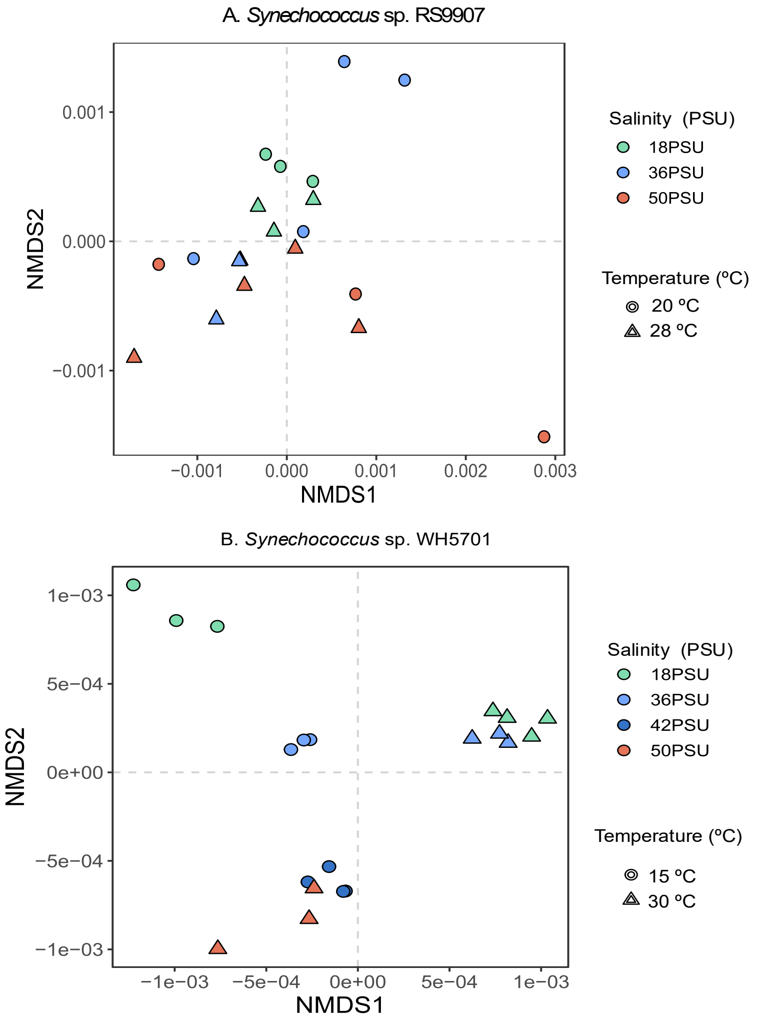

Figure S4. Non multidimensional scaling plot of RNA samples obtained during the acclimation to salinity and temperature of *Synechococcus* sp. RS9907 (A) and WH5701 (B). Green, light blue, dark blue and red colors refer to salinity samples, 18, 36, 42 and 50 PSU, respectively. Circle and triangle shapes indicate the temperature of acclimation in both strains. The ENVFIT analysis indicates that salinity significantly influences the transcriptional ordination in RS9907, while in WH5701, both salinity and temperature influence the transcriptional ordination ($p < 0.05$).

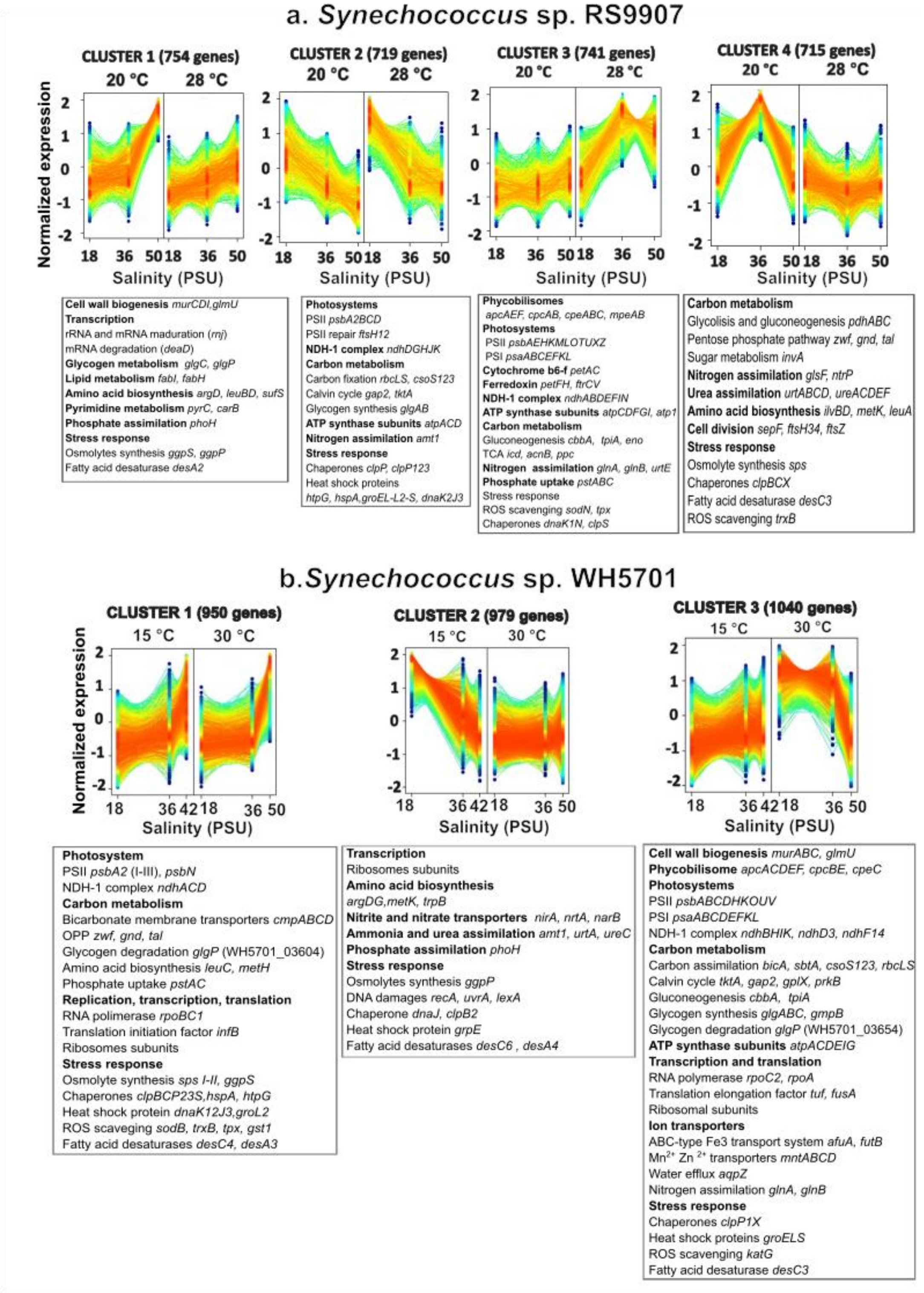

Figure S5. Functional genes identified in each of the clusters based on expression profiles across different salinity and temperature conditions of *Synechococcus* sp. RS9907 (a) and *Synechococcus* sp. WH5701 (b). For each cluster, the normalized expression values are shown, within right and left panels indicate both temperatures of acclimation, 20 °C and 28 °C in RS9907 and 15 °C and 30 °C in WH5701, respectively. The colours of the dots and lines indicate the membership value assigned by the fuzzy c-means soft clustering of each gene. The values range from 1 (red, indicating a high score) to 0.5 (blue, indicating a low score). The total number of genes assigned to each cluster is shown. TCA refers to tricarboxylic acid cycle genes and OPP indicate oxidative pentose phosphate pathway genes.

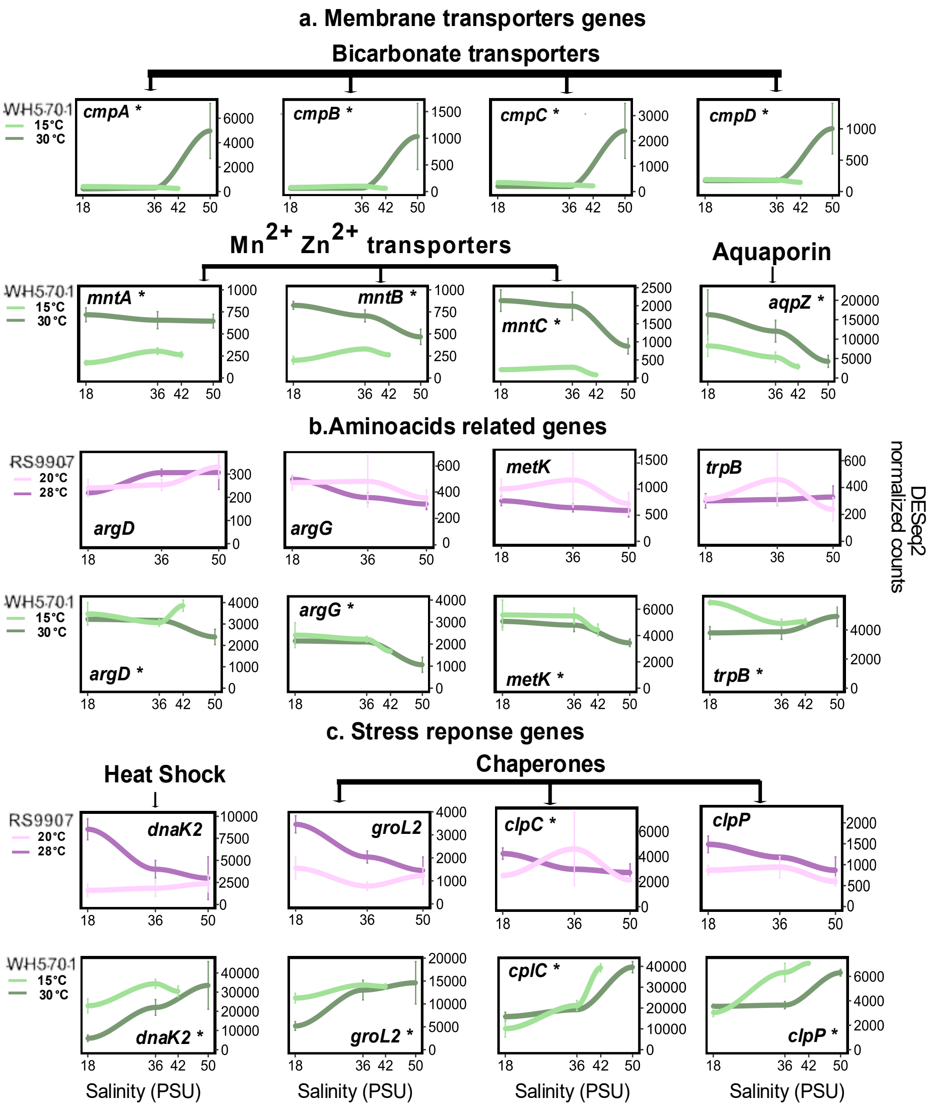

Figure S6. Gene expression values of a selection of individual genes in both *Synechococcus* strains along the salinity gradient and at two growth temperatures conditions. (a) Membrane transporters genes only identified in WH5701 genome, (b) Genes involved in amino acid metabolism, (c) Genes related to stress response. At each salinity, the average of three biological replicates is shown, and error bars represent the standard deviation. Asterisks denote differential expression along the salinity gradient (LTR test, α = 0.01). Light colours refer to low temperatures (20ºC and 15ºC for RS9907 and WH5701, respectively) and dark colours refer to optimal growth temperatures (28ºC and 30ºC for RS9907 and WH5701, respectively). RS9907 values appear in pink and WH5701 values appear in green.
